# Rice seed priming with sodium selenate: Effects on germination, seedling growth, and biochemical attributes

**DOI:** 10.1038/s41598-019-40849-3

**Published:** 2019-03-13

**Authors:** Bin Du, Haowen Luo, Longxin He, Lihe Zhang, Yangfang Liu, Zhaowen Mo, Shenggang Pan, Hua Tian, Meiyang Duan, Xiangru Tang

**Affiliations:** 1 Department of Crop Science and Technology, College of Agriculture, South China Agricultural University, Guangzhou, 510642 China; 2 Scientific Observing and Experimental Station of Crop Cultivation in South China, Ministry of Agriculture, Guangzhou, 510642 China

## Abstract

The aim of this study was to determine the effects of sodium selenate (15, 30, 45, 60, 75, 90, and 105 mg kg^−1^) on the germination and seedling growth of Changnongjing 1 rice (*Oryza sativa* L.) at 25 °C and 30 °C. Low selenate concentrations induced shorter and more uniform germination periods than did ultrapure water at both temperatures. Seedlings primed with low selenate concentrations were superior to those primed with ultrapure water in terms of plant height, fresh weight, dry matter accumulation, and soluble carbohydrate and protein contents. Lower selenate concentrations (15–75 mg kg^−1^) induced higher chlorophyll and phenol contents in seedlings than did ultrapure water. Lower selenate concentrations also increased the superoxide dismutase (SOD), peroxidase (POX), catalase (CAT), and glutathione peroxidase (GPx) contents in seedlings and significantly decreased the stress-related malondialdehyde (MDA) content compared to ultrapure water. In conclusion, rice seedling germination and growth were promoted by priming with low selenate concentrations (15–75 mg kg^−1^) but inhibited by priming with high selenate concentrations (90–105 mg kg^−1^).

## Introduction

With the development of intensive agriculture worldwide, transplant cultivation has become a limiting factor. Direct-seeded cultivation is an alternative to transplanting. This technique accelerates soil preparation and reduces labour and water costs by 20% and 30%, respectively, compared to transplanting^1^. The seeds used in direct-seeded cultivation must have higher germination rates and uniformity than those used in transplanting. Seeds sown directly may be subjected to low temperatures, drought, flooding, and other adverse conditions. Embryonic cell membrane damage results in severe extracellular solute infiltration, lower seed moisture and vitality, and the accumulation of reactive oxygen species (ROS). All these factors reduce the seed germination rate. At low temperatures, however, antioxidants such as peroxidase (POD), catalase (CAT), and glutathione peroxidase (GPx) in the seeds scavenge free radicals. In this way, these enzymes protect the plants by alleviating low-temperature chilling effects. Seed initiation can increase the antioxidant enzyme content and vigour of seeds. Seed priming can also enhance antioxidant levels and the activity of oxidative defensive enzymes^2,3^. Certain trace elements are also known as seed initiators. When selenium (Se) is used as a seed initiator, it increases seed activity and vitality and serves as a human dietary source of this essential micronutrient^4^. Selenium is absorbed during rice germination and improves germination quality^5,6^, root activity^7^, and seedling growth and vitality^8^. This element also ensures high germination quality of seeds for direct rice seeding. Selenium also increases the antioxidant and defensive substance contents and activities in plants^2^, provides energy for germination and accelerates phosphorylation during membrane formation^3^. Selenium improves stress resistance and antioxidant and enzyme activity in plants. Plant selenium levels are closely related to human dietary selenium status^9^. Low selenium levels in humans are a global public health concern. Insufficient selenium intake can induce epilepsy, reduce fertility, and cause immunodeficiency^10–12^. Selenium has anti-ageing, anticancer, and immunity-boosting effects in humans. Artificial selenium supplementation is a basic biofortification strategy^13^. This strategy optimizes crop yield and quality and increases selenium levels in the human body^14^. Although selenium can improve seed germination and seedling quality, sodium selenate is rarely used as a seed initiator. Sodium selenite is known to initiate and prime seeds^3^. In the present study, we assume that sodium selenate can also ensure the uniformity of germination. Our objective was to investigate the application of sodium selenate as a seed germination initiator at different temperatures.

## Results

### Emergence attributes

The various concentrations of sodium selenate at the two temperatures significantly affected rice seed germination characteristics (*P* ≤ 0.05; Table 1) and the time to start of emergence (TSE) (*P* < 0.05). Nevertheless, the relative sensitivity of the TSE to the various sodium selenate treatments differed between the two temperatures. The TSE was advanced by 2 d after treatment with 60 mg kg^−1^ sodium selenate at 25 °C and 45 mg kg^−1^ sodium selenate at 30 °C. After treatment with > 60 mg kg^−1^ sodium selenate, the TSE was significantly delayed at both temperatures. Sodium selenate significantly affected the final emergence percentage (FEP) on day 7 (*P* ≤ 0.05). The TSE did not significantly differ between the 25 °C and 30 °C treatments. On day 7, the germination potential increased with an increase in sodium selenate concentration from 15–45 mg kg^−1^. At 45 mg kg^−1^ sodium selenate, the germination potential reached a maximum. When the concentration exceeded 75 mg kg^−1^, the germination potential tended to decline. The same pattern was observed for the FEP. Sodium selenate concentration significantly affected the FEP (*P* ≤ 0.05), but there were no significant differences between 25 and 30 °C. However, at both temperatures, the FEP reached a maximum at 60 mg kg^−1^ sodium selenate. Therefore, low-concentration selenate treatments promoted Changnongjing 1 rice seed germination, whereas high-concentration selenate treatments inhibited it.

**Table 1:** Effects of selenium seed priming on rice germination.

Treatment	TSE			Day 7 FEP			FEP		
	25 °C	30 °C	Mean	25 °C	30 °C	Mean	25 °C	30 °C	Mean
0	2.67 ± 0.33AB	2.67 ± 0.33AB	2.67 ± 0.30AB	48.00 ± 4.99BCD	48.67 ± 8.67BC	48.33 ± 4.74CD	67.33 ± 0.54CD	68.67 ± 2.40D	68.00 ± 1.15EF
15	2.67 ± 0.33AB	2.67 ± 0.33AB	2.67 ± 0.30AB	48.67 ± 3.31BCD	52.00 ± 2.00BC	50.33 ± 2.16BC	70.67 ± 1.44BCD	75.33 ± 1.33C	73.00 ± 1.44DE
30	2.33 ± 0.33AB	2.67 ± 0.33AB	2.50 ± 0.32AB	56.67 ± 1.96ABC	58.00 ± 4.62AB	57.33 ± 2.35AB	77.33 ± 1.44B	80.00 ± 2.31BC	78.67 ± 1.43BC
45	2.67 ± 0.33AB	2.00 ± 0.00B	2.33 ± 0.30AB	62.67 ± 1.96ABC	69.33 ± 4.67A	66.00 ± 2.78A	78.67 ± 1.44B	84.00 ± 1.15AB	81.33 ± 1.52B
60	2.00 ± 0.00B	2.33 ± 0.33AB	2.17 ± 0.24B	59.33 ± 1.09ABC	61.33 ± 2.40AB	60.33 ± 1.31A	87.33 ± 1.09A	86.67 ± 0.67A	87.00 ± 0.68A
75	3.00 ± 0.00A	2.67 ± 0.33AB	2.83 ± 0.24A	50.67 ± 1.44ABCD	49.33 ± 6.36BC	50.00 ± 2.97BC	75.33 ± 1.44BC	75.33 ± 1.33C	75.33 ± 0.99CD
90	2.67 ± 0.33AB	2.67 ± 0.33AB	2.67 ± 0.30AB	45.33 ± 5.99CD	47.33 ± 5.33BC	46.33 ± 4.08CD	63.33 ± 1.96D	68.00 ± 1.15D	65.67 ± 1.58FG
105	2.67 ± 0.33AB	3.00 ± 0.00A	2.83 ± 0.24A	42.67 ± 5.52D	37.33 ± 1.76C	40.00 ± 3.35D	65.33 ± 5.36D	58.67 ± 3.53E	62.00 ± 3.65G

Means with different letters differ significantly at the 5% probability level based on Tukey’s test.

The various sodium selenate concentrations significantly affected the Changnongjing 1 germination indices (*P* ≤ 0.05) at both temperatures. The effects of sodium selenate concentration on Changnongjing 1 priming at 25 °C and 30 °C are shown in Table 2. Clearly, the E_50_ varied significantly with sodium selenate concentration. Nevertheless, E_50_ did not significantly vary with temperature. At both 25 °C and 30 °C, the E_50_ decreased with increasing sodium selenate concentration from 15–75 mg kg^−1^, reaching a minimum at 45 mg kg^−1^. However, >75 mg kg^−1^ sodium selenate prolonged germination time by 50%. Temperature and sodium selenate concentration significantly affected the germination index. The germination index was highest at a temperature of 30 °C and a sodium selenate concentration of 45 mg kg^−1^. At both temperatures, the germination index increased with an increase in sodium selenate concentration from 15–75 mg kg^−1^ but decreased at >90 mg kg^−1^ sodium selenate. The germination index represents the speed and uniformity of germination. After receiving 45 mg kg^−1^ sodium selenate, the Changnongjing 1 rice seeds germinated rapidly and uniformly at both 25 °C and 30 °C (Table 2).

**Table 2:** Effects of selenium seed priming on germination attributes of rice.

Treatment	E_50_			MET			EI		
	25 °C	30 °C	Mean	25 °C	30 °C	Mean	25 °C	30 °C	Mean
Se_0_	5.66 ± 0.24AB	5.66 ± 0.57AB	5.66 ± 0.29B	5.80 ± 0.23A	5.74 ± 0.29AB	5.81 ± 0.18ABC	6.94 ± 0.57BC	7.31 ± 0.52D	7.12 ± 0.40CD
Se_15_	5.55 ± 0.22B	5.14 ± 0.42BC	5.34 ± 0.24BC	6.06 ± 0.09A	5.52 ± 0.18B	5.79 ± 0.15ABC	6.72 ± 0.25BC	7.99 ± 0.24CD	7.35 ± 0.34BCD
Se_30_	5.31 ± 0.18BC	4.60 ± 0.170CD	4.95 ± 0.20C	5.76 ± 0.08A	5.34 ± 0.04BC	5.55 ± 0.11C	7.81 ± 0.21AB	8.85 ± 0.38BC	8.33 ± 0.31BCD
Se_45_	4.37 ± 0.10C	3.99 ± 0.21D	4.18 ± 0.14D	5.27 ± 0.08A	4.83 ± 0.14C	5.05 ± 0.12D	8.84 ± 0.12A	10.43 ± 0.40A	9.64 ± 0.40A
Se_60_	5.13 ± 0.10BC	4.49 ± 0.26CD	4.81 ± 0.19CD	5.76 ± 0.06A	5.29 ± 0.17BC	5.53 ± 0.13C	9.02 ± 0.22A	9.97 ± 0.47AB	9.50 ± 0.32A
Se_75_	5.79 ± 0.44AB	4.98 ± 0.19BC	5.39 ± 0.31BC	5.81 ± 0.05A	5.53 ± 0.14B	5.67 ± 0.09BC	7.79 ± 0.09AB	7.98 ± 0.09CD	7.89 ± 0.08BC
Se_90_	5.90 ± 0.31AB	5.83 ± 0.39AB	5.87 ± 0.25A	6.00 ± 0.20A	5.95 ± 0.39AB	5.97 ± 0.21AB	6.33 ± 0.45C	6.85 ± 0.59D	6.59 ± 0.38DE
Se_105_	6.69 ± 0.43A	6.29 ± 0.05A	6.49 ± 0.25AB	5.98 ± 0.07A	6.35 ± 0.25AB	6.17 ± 0.14A	6.55 ± 0.62BC	5.43 ± 0.51E	5.99 ± 0.48E

Means with different letters differ significantly at the 5% probability level based on Tukey’s test. Subscript numbers after Se denote the selenium concentrations (μmol L^−1^) used for seed priming.

### Seedling growth

Sodium selenate also had a strong positive effect on rice seedling growth. Seedlings developed from seeds germinated with sodium selenate were of higher quality than those primed with water alone (Table 3). Sodium selenate significantly affected rice plant height (Table 3). When the selenate concentration was >45 mg kg^−1^, the plant height began to decrease until 75 mg kg^−1^. At this high dose, the plants were shorter than those derived from seeds initiated with plain water. At all selenate concentrations, the plants subjected to 25 °C were taller than those exposed to 30 °C. Sodium selenate significantly affected the shoot fresh weight (*P* ≤ 0.05) (Table 3). Shoot fresh weights were greater in the 45, 60, and 75 mg kg^−1^ selenate treatments than in the water controls and lower in the 15, 90, and 105 mg kg^−1^ selenate treatments than in the controls. The maximum shoot fresh weight was obtained at 45 mg kg^−1^ sodium selenate. At 30 °C, the shoot fresh weights at all selenate concentrations except 105 mg kg^−1^ were greater than those in the water controls. The maximum shoot fresh weight was recorded at 45 mg kg^−1^ sodium selenate. The shoot fresh weights at 25 °C were significantly higher than those at 30 °C (*P* ≤ 0.05). Therefore, only certain sodium selenate concentrations and a temperature of 25 °C were conducive to rice seedling growth. Sodium selenate also significantly affected the shoot dry weight. Shoot dry matter accumulation in rice seedlings significantly varied with selenate concentration at 25 °C. Shoot dry matter accumulation increased with selenate concentration between 15 and 70 mg kg^−1^, peaked at 45 mg kg^−1^, and decreased from 75–105 mg kg^−1^. In contrast, at 30 °C, the shoot dry matter accumulation did not significantly differ between any selenate doses and the water control. Nevertheless, the shoot dry matter increased with an increase in sodium selenate concentration from 15–60 mg kg^−1^, peaked at 45 mg kg^−1^, and decreased between 7–105 mg kg^−1^. These patterns parallel those observed at 25 °C. Overall, the accumulation of shoot dry matter in rice seedlings did not significantly differ between 25 °C and 30 °C.

**Table 3:** Effects of selenium seed priming on aboveground growth of rice seedlings.

Treatment	Plant height			Fresh weight of aerial part			Dry matter weight of aerial part		
	25 °C	30 °C	Mean	25 °C	30 °C	Mean	25 °C	30 °C	Mean
Se_0_	6.37 ± 0.20D	5.38 ± 0.11D	5.88 ± 0.34D	43.48 ± 2.91B	35.09 ± 3.78C	39.28 ± 4.02B	5.66 ± 0.36C	4.7 ± 0.68A	5.18 ± 0.57D
Se_15_	6.52 ± 0.01D	5.53 ± 0.12D	6.03 ± 0.32D	42.16 ± 1.22B	34.79 ± 0.53C	38.47 ± 2.48B	6.62 ± 0.47ABC	5.21 ± 0.19A	5.91 ± 0.55BCD
Se_30_	7.14 ± 0.27C	5.81 ± 0.17D	6.48 ± 0.47CD	42.85 ± 1.00A	35.35 ± 2.26BC	39.1 ± 2.84B	6.70 ± 0.12ABC	4.93 ± 0.23A	5.82 ± 0.58BCD
Se_45_	8.55 ± 0.10A	7.72 ± 0.08A	8.17 ± 0.27A	53.56 ± 3.12A	55.20 ± 3.25A	54.38 ± 2.90A	7.02 ± 0.37ABC	6.92 ± 0.60A	6.97 ± 0.45AB
Se_60_	7.67 ± 0.15B	7.41 ± 0.05AB	7.54 ± 0.13AB	54.72 ± 0.16A	45.02 ± 4.28ABC	49.87 ± 4.09A	7.39 ± 0.38AB	7.04 ± 1.79A	7.22 ± 1.16A
Se_75_	7.34 ± 0.16BC	6.84 ± 0.06BC	7.09 ± 0.19BC	50.91 ± 0.22B	46.55 ± 1.62AB	48.73 ± 1.72A	6.79 ± 0.48A	6.85 ± 0.19A	6.82 ± 0.33AB
Se_90_	6.06 ± 0.05D	6.58 ± 0.33C	6.32 ± 0.27D	39.14 ± 2.44B	42.76 ± 5.88BC	40.95 ± 4.19B	6.82 ± 0.40AB	6.39 ± 0.55A	6.60 ± 0.45ABC
Se_105_	6.26 ± 0.23D	5.47 ± 0.38D	5.87 ± 0.38D	42.31 ± 2.34B	36.16 ± 5.43BC	39.24 ± 4.21B	6.18 ± 0.33BC	4.89 ± 0.67A	5.53 ± 0.63CD

Means with different letters differ significantly at the 5% probability level based on Tukey’s test. Subscript numbers after Se denote the selenium concentrations (μmol L^−1^) used for seed priming.

The effects of sodium selenate on rice seedling root development are shown in Table 4. Neither selenate concentration nor temperature significantly affected rice seedling root length. Selenate did, however, significantly affect root fresh weight (*P* ≤ 0.05). At 25 °C, the root fresh weights for all selenate treatments except 30 mg kg^−1^ were greater than those for the water control. The root fresh weight was highest in the 60 mg kg^−1^ treatment. At 30 °C, however, the root fresh weights for all selenate treatments except 15 mg kg^−1^ and 75 mg kg^−1^ surpassed those for the water control. At 30 °C, the maximum root fresh weight was measured in the 60 mg kg^−1^ sodium selenate treatment. The root fresh weights at 30 °C were significantly higher than those at 25 °C (*P* ≤ 0.05). Thus, rice seedling roots grew better at 30 °C. Sodium selenate significantly affected the rice root dry weight (Table 3). The greatest root dry weights at 25 °C and 30 °C were recorded for the 60 mg kg^−1^ and 75 mg kg^−1^ sodium selenate treatments, respectively. The root dry weights also significantly differed between 25 °C and 30 °C.

**Table 4:** Effects of selenium seed priming on belowground growth of rice seedlings.

Treatment	Root length			Fresh weight of aerial part			Dry matter weight of aerial part		
	25 °C	30 °C	Mean	25 °C	30 °C	Mean	25 °C	30 °C	Mean
Se_0_	8.00 ± 0.99ABC	8.49 ± 1.39AB	8.25 ± 1.09A	107.05 ± 5.33D	114.24 ± 7.35AB	110.65 ± 6.18B	5.61 ± 0.44B	5.72 ± 0.61B	5.67 ± 0.48AB
Se_15_	6.10 ± 0.06C	7.62 ± 0.39AB	6.86 ± 0.54A	113.66 ± 1.41CD	115.87 ± 0.78AB	114.77 ± 1.24B	6.65 ± 0.09B	6.53 ± 0.23B	6.59 ± 0.16AB
Se_30_	7.19 ± 0.16BC	8.70 ± 0.65AB	7.95 ± 0.64A	103.45 ± 5.50D	113.57 ± 3.76AB	108.51 ± 5.29B	6.09 ± 0.4B	6.02 ± 0.56B	6.05 ± 0.44AB
Se_45_	8.97 ± 0.58AB	8.71 ± 1.17AB	8.84 ± 0.83A	118.63 ± 1.17BCD	128.94 ± 8.16A	123.79 ± 6.15AB	5.91 ± 0.23B	6.39 ± 0.37B	6.15 ± 0.31AB
Se_60_	6.49 ± 0.49C	8.19 ± 0.42AB	7.34 ± 0.68A	141.38 ± 5.63A	134.46 ± 13.40A	137.92 ± 9.45A	6.78 ± 0.04B	12.83 ± 5.54A	9.81 ± 3.99AB
Se_75_	9.15 ± 0.95A	6.01 ± 0.84B	7.58 ± 1.28A	132.01 ± 5.02AB	95.39 ± 1.99B	113.70 ± 12.07B	14.67 ± 6.46A	5.35 ± 0.57B	10.01 ± 5.05A
Se_90_	6.27 ± 0.52C	9.98 ± 1.27A	8.13 ± 1.46A	123.48 ± 3.31BC	118.76 ± 13.28AB	121.12 ± 8.78AB	5.38 ± 0.21B	5.23 ± 0.89B	5.31 ± 0.58B
Se_105_	6.63 ± 0.75C	7.50 ± 1.44AB	7.06 ± 1.06A	112.4 ± 8.82CD	111.22 ± 21.51AB	111.81 ± 14.71B	6.28 ± 0.45B	5.44 ± 0.93B	5.86 ± 0.71AB

Means with different letters differ significantly at the 5% probability level based on Tukey’s test. Subscript numbers after Se denote the selenium concentrations (μmol L^−1^) used for seed priming.

### Biochemical attributes

**Figure 1:**
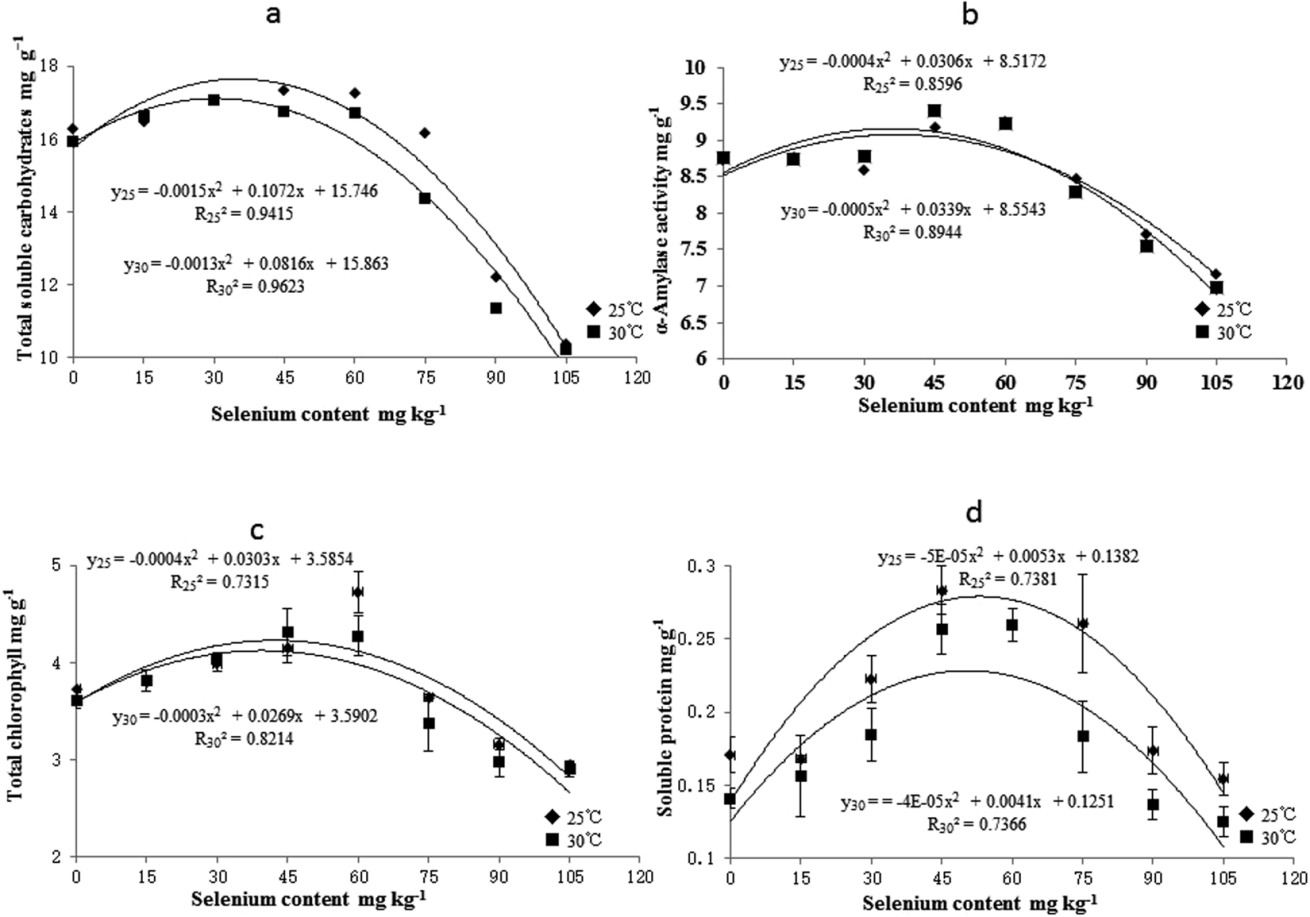
Effects of selenium priming on Total soluble carbohydrates α-mylase activity Total chlorophyll and Soluble protein in rice seedlings at different temperatures. Vertical bars above and below each mean denote the standard error of six replicates.

Sodium selenate concentration and temperature both significantly affected the total soluble carbohydrate content in rice seedlings (Fig. 1a). The total soluble carbohydrate content varied with selenate concentration (y_25_ = −0.0015x^2^ + 0.1072x + 15.746; y_30_ = −0.0013x^2^ + 0.0816x + 15.863). The maximum total soluble carbohydrate content was detected at 45 mg kg^−1^ selenate. The total soluble carbohydrate content was higher within the 15–60 mg kg^−1^ selenate concentration range and lower when the selenate concentration was >75 mg kg^−1^ than in the water control. Selenate also significantly affected the α-amylase activity in rice seedlings. The α-amylase activity varied quadratically with selenate concentration and temperature (y_25_ = −0.0004x^2^ + 0.0306x + 8.5172; y_30_ = −0.0005x^2^ + 0.0339x + 8.5543) (Fig. 1b). The maximum α-amylase activity was observed at 45 mg kg^−1^ selenate. The α-amylase activity was higher within the 15–60 mg kg^−1^ selenate concentration range and lower when the selenate concentration was >75 mg kg^−1^ than in the water control.

Sodium selenate significantly affected the total chlorophyll content in rice seedlings. The total chlorophyll content varied quadratically with temperature and selenate concentration (y_25_ = −0.0004x^2^ + 0.0303x + 3.5854; y_30_ = −0.0003x^2^ + 0.0269x + 3.5902) (Fig. 1c). The peak chlorophyll content was measured in the 45 mg kg^−1^ selenate treatment. The chlorophyll content was greater within the 15–60 mg kg^−1^ selenate concentration range and lower at 7 mg kg^−1^ selenate than in the water control. Selenate also significantly affected the water-soluble protein content of rice seedlings. The water-soluble protein content varied quadratically with selenate concentration and temperature (y_25_ = 5E-05x^2^ + 0.0053x + 0.1382; y_30_ = −4E-05x^2^ + 0.0041x + 0.1251) (Fig. 1d). The maximum water-soluble protein content was found in the 45 mg kg^−1^ selenate treatment.

**Figure 2:**
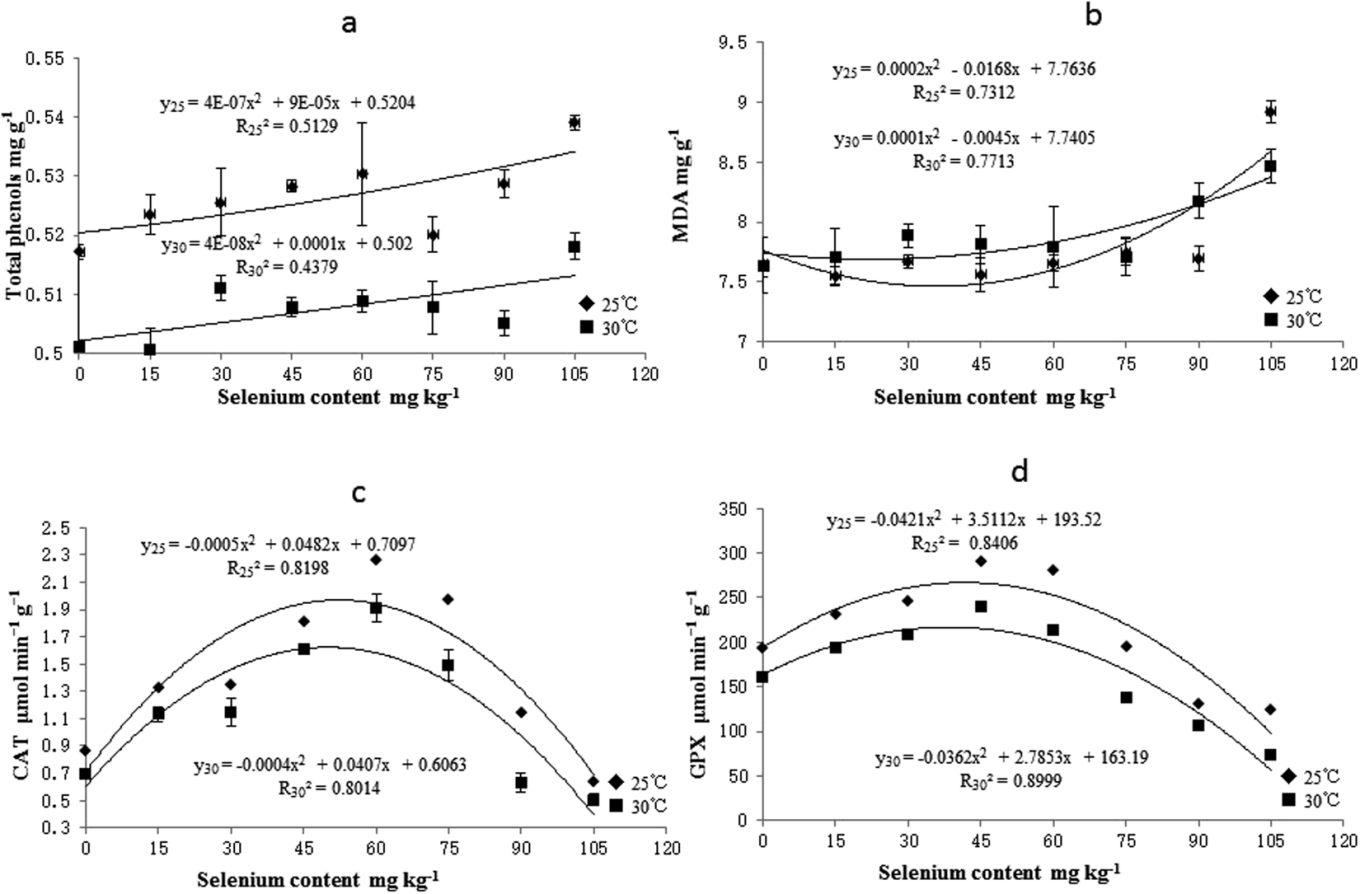
Effects of selenium priming on Total phenols MDA CAT and GPx in rice seedlings at different temperatures. Vertical bars above and below each mean denote the standard error of six replicates.

Sodium selenate significantly affected the total phenol content of the rice seedlings. The total phenol content varied quadratically with selenate concentration and temperature (y_25_ = y = 4E-07x^2^ + 9E-05x + 0.5204; y_30_ = y = 4E-08x^2^ + 0.0001x + 0.502) (Fig. 2a). The total phenol content increased with selenate concentration. Selenate also significantly affected the MDA levels in rice seedlings. The MDA content varied quadratically with selenate concentration and temperature (y_25_ = y = 0.0002x^2^ − 0.0168x + 7.7636; y_30_ = 0.0001x^2^ − 0.0045x + 7.7405) (Fig. 2b). The lowest MDA levels were detected for the 45 mg kg^−1^ selenate treatment. The MDA content was lower in the 15–75 mg kg^−1^ selenate concentration range and higher at >5 mg kg^−1^ selenate than in the water control.

Sodium selenate significantly affected the CAT activity in rice seedlings. CAT activity varied quadratically with selenate concentration and temperature (y_25_ = −0.0005x^2^ + 0.0482x + 0.7097; y_30_ = −0.0004x^2^ + 0.0407x + 0.6063) (Fig. 2c). The maximum CAT activity was observed for the 45 mg kg^−1^ selenate treatment. The CAT activity was higher within the 15–75 mg kg^−1^ selenate concentration range and lower at >75 mg kg^−1^ selenate than in the water control. GPx activity also varied quadratically with selenate concentration and temperature (y_25_ = −0.0421x^2^ + 3.5112x + 193.52; y_30_ = −0.0362x^2^ + 2.7853x + 163.19) (Fig. 2d). GPx activity peaked at 45 mg kg^−1^ selenate. The GPx activity was higher within the 15–75 mg kg^−1^ selenate concentration range and lower at >75 mg kg^−1^ selenate than in the water control.

**Figure 3:**
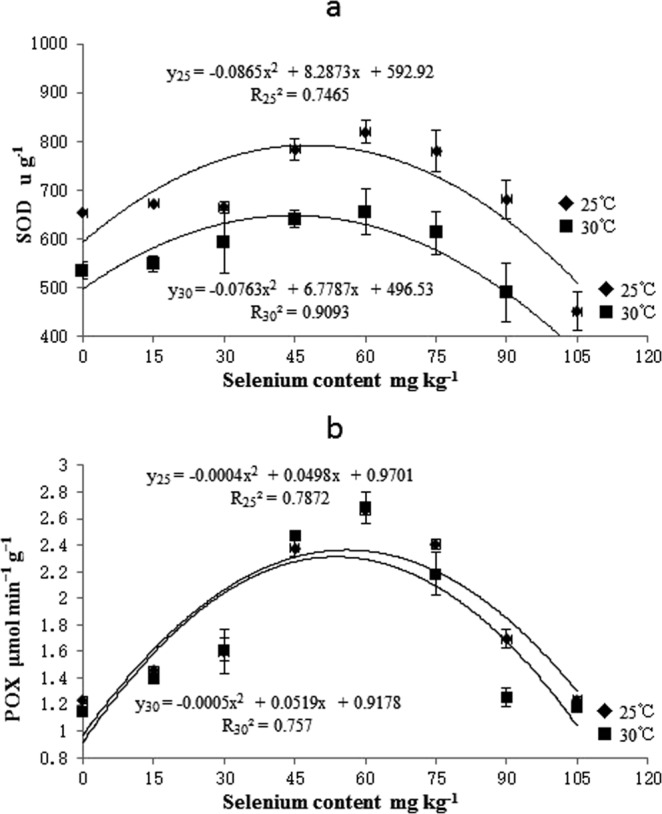
Effects of selenium seed priming on SOD and POX of rice seedlings at different temperatures. Vertical bars above and below each mean denote the standard error of six replicates.

Sodium selenate significantly affected the SOD in rice seedlings. SOD activity varied quadratically with selenate concentration and temperature (y_25_ = −0.0865x^2^ + 8.2873x + 592.92; y_30_ = −0.0763x^2^ + 6.7787x + 496.53) (Fig. 3a). SOD activity reached a maximum at 45 mg kg^−1^ selenate. The SOD activity was higher within the 15–75 mg kg^−1^ selenate concentration range and lower at >75 mg kg^−1^ selenate than in the water control. Selenate also significantly affected the POX activity in rice seedlings. POX activity varied quadratically with selenate concentration and temperature (y_25_ = −0.0004x^2^ + 0.0498x + 0.9701; y_30_ = −0.0005x^2^ + 0.0519x + 0.9178) (Fig. 3b). The POX activity peaked at 45 mg kg^−1^ selenate. The POX activity was higher within the 15–75 mg kg^−1^ selenate concentration range and lower at >75 mg kg^−1^ selenate than in the water control.

## Discussion

Selenium is not considered an essential trace element (micronutrient) for the growth and development of higher plants^15^. Nevertheless, this element has positive effects on many crops such as potato (*Solanum tuberosum* L.), ryegrass (*Lolium perenne* L.), lettuce (*Lactuca sativa* L.), and mustard (*Brassica rapa* L.)^7,16,17^. The present study showed that low concentrations of sodium selenate act as seed priming initiators and are beneficial for plants. Khaliq *et al*.^6^ studied the effect of sodium selenite on Basmati rice germination and seedling growth. Moulick *et al*.^18^ investigated the effects of selenium on the germination of rice seeds under heavy metal stress. In both cases, however, selenium was supplied in the form of sodium selenite instead of sodium selenate. In the present study, sodium selenate was used for seed priming. Low-concentration sodium selenate treatments had positive effects on seed germination mainly by optimizing germination characteristics such as the TSE, E_50_, MET, EI, and FEP. Selenate at 45–60 mg kg^−1^ significantly outperformed water and higher selenate concentrations as a seed germination initiator. In contrast, very high selenate levels inhibited germination. This finding corroborated those reported by Khaliq *et al*.^6^. Compared to the water control and the high-dose selenate treatments, low selenate concentrations significantly increased plant height, root length, and shoot and root biomass accumulation. Therefore, low selenate concentrations significantly affect plant growth ability. However, high selenate doses inhibited seedling growth and development, corroborating the data of Moulick *et al*.^18^. Low selenate concentrations are thus preferable for seed germination and seedling development^19^. The use of low levels of sodium selenate can increase the germination rate and uniformity in direct-seeded rice. Such levels of sodium selenate can also improve overall seedling quality. Relative to the water control, low selenate doses improved dry matter accumulation in rice seedlings. In contrast, high selenate concentrations actually inhibited seedling dry matter accumulation. This negative effect may be explained by the inhibitory influence of selenate on chlorophyll content and α-amylase activity. There is no evidence that sodium selenate directly participates in higher plant photosynthesis. Nevertheless, photosynthetic activity in rice is closely correlated with sodium selenate application^20^. Low selenate concentrations may promote photosynthesis^21,22^ and increase the chlorophyll content^18^ and α-amylase activity^3^ in rice. High sodium selenate concentrations have the opposite effects. The present study shows that there is a quadratic relationship between soluble sugar/soluble protein content and selenate concentration. Similarly, Lyons *et al*.^16^ found that low selenate concentrations increased photosynthesis. The total phenol content increased with selenate concentration, but the correlation was not significant at the early stages of seedling development. At >75 mg kg^−1^ selenate, the seedling phenol content significantly increased. The rice seedlings were abiotically stressed at these very high selenate concentrations. MDA is another important biochemical indicator of plant stress. MDA levels increase quadratically with selenate concentration. MDA was lowest at 45 mg kg^−1^ selenate. The MDA content was lower within the 15–75 mg kg^−1^ selenate concentration range and higher at >75 mg kg^−1^ selenate than in the water control. Since sodium selenate is also an antioxidant^23^, the reduced MDA content at low selenate concentrations may be correlated with selenate antioxidant activity. Relative to the water control, however, >75 mg kg^−1^ selenate increased the MDA content. Therefore, very high selenate levels may be phytotoxic, as the high seedling phenol content at these selenate doses can have negative impacts on rice seedlings; this finding is consistent with the result for phenol content. The changes in CAT, GPx, SOD, and POX activity were also consistent with selenate antioxidant activity. Peroxide and free radicals formed during seed germination and seedling development are scavenged and quenched by a series of antioxidant enzymes^24,25^. Khaliq *et al*.^6^ reported similar observations using sodium selenite as a seed initiator.

As a seed germination initiator, 15–75 mg kg^−1^ sodium selenate can increase rice seed germination and enhance seedling growth. Low selenate doses may also increase starch, protein and sugar accumulation in the seedlings and improve their antioxidant capacity. The accumulation of sodium selenate in rice should also be assessed as a biofortification mechanism.

## Methods

In this study, the rice (*Oryza sativa* L.) variety Changnongjing 1 (Yangtze University, Hubei, China) was used. The initial seed moisture content was 9.22%. Dry rice seeds were primed at two different temperatures (25 °C or 30 °C) in a solution of sodium selenate (Na_2_O_4_Se) at a 0, 15, 30, 45, 75, 90, or 105 mg kg^−1^ concentration. A two-factor, completely randomized design was used. In each treatment, 25 g of seeds was placed in a 200-mL conical flask containing 125 mL initiator solution^26^. The flasks were placed in an incubator [darkness, 25 ± 1 °C or 30 ± 1 °C, and 80% relative humidity]. The flasks were shaken once every 6 h^27^. After priming, the seeds were filtered through gauze, placed in distilled water for 20 min, rinsed five times with ultrapure water and set aside. Autoclavable glass Petri dishes were lined with double layers of filter paper, placed on a laboratory bench and left to air dry for 24 h^3^.

The Petri dishes were sterilized by autoclaving and left to cool. The seeds were surface sterilized by soaking for 15 Min in 5% sodium hypochlorite and washed with ultrapure water 3–5 times. Thereafter, 10 mL sterile water was aseptically pipetted into each Petri dish; then, 100 seeds were placed in each Petri dish, and each seed-soaking treatment was performed in triplicate. The Petri dishes were transferred into a phytotron (KRG-400BP, China) and incubated under 8,000 lx illumination for 14 h d^−1^. No light was used for the remaining 10 h. The temperature was either 25 ± 1 °C or 30 ± 1 °C, and the relative humidity was 80%^28^. The Petri dishes were weighed daily and replenished with the required amount of selenate solution.

### Seed bioassay

Seedling emergence was counted daily using the method prescribed by the Association of Official Seed Analysts (AOSA)^29^ until a constant count was reached. A seedling was scored as emerged when the hypocotyl length was ≥2 mm. The time to 50% emergence (*E*_50_) was calculated according to the modified equation of Farooq *et al*.^30^:

$${E}_{{\rm{50}}}=ti+\frac{(\frac{N}{2}-ni)(tj-ti)}{nj-ni}$$

1 $${{\rm{E}}}_{50}={{\rm{t}}}_{{\rm{i}}}+[({\rm{N}}/2-{{\rm{n}}}_{{\rm{i}}})({{\rm{t}}}_{{\rm{j}}}-{{\rm{t}}}_{{\rm{i}}})]/({{\rm{n}}}_{{\rm{j}}}-{{\rm{n}}}_{{\rm{i}}})$$

where *N* is the final number of emerged seeds; *n*_i_ and *n*_j_ are the cumulative number of emerged seeds counted at time *t*_i_ and *t*_j_, respectively, where *n*_i_ < *N*/2 < *n*_j_.

The mean emergence time (MET) was calculated following Ellis and Roberts^31^:

2 $${\rm{MET}}={\rm{\Sigma }}\mathrm{Dn}/{\rm{\Sigma }}n$$

where *n* is the number of emerged seeds on day *D*, and *D* is the number of days counted from the beginning of emergence.

$${\rm{MET}}=\frac{\sum Dn}{\sum n}$$

The emergence index (EI) was calculated as described by the AOSA^32^:

3 $$\begin{array}{c}{\rm{EI}}=[({\rm{Number}}\,{\rm{of}}\,{\rm{emerged}}\,{\rm{seeds}})/({\rm{days}}\,{\rm{of}}\,{\rm{first}}\,{\rm{count}})]+\ldots \\ \,\,\,\,\,+\,[({\rm{Number}}\,{\rm{of}}\,{\rm{emerged}}\,{\rm{seeds}})/({\rm{days}}\,{\rm{of}}\,{\rm{first}}\,{\rm{count}})]\end{array}$$

$${\rm{EI}}=\frac{{\rm{No}}.\,{\rm{of}}\,{\rm{emerged}}\,{\rm{seeds}}}{{\rm{Days}}\,{\rm{of}}\,{\rm{first}}\,{\rm{count}}}+\ldots \ldots +\frac{{\rm{No}}.\,{\rm{of}}\,{\rm{emerged}}\,{\rm{seeds}}}{{\rm{Days}}\,{\rm{of}}\,{\rm{final}}\,{\rm{count}}}$$

The root and shoot lengths of five seedlings randomly selected from each experimental unit were measured 18 d after sowing (DAS). The roots and shoots were excised separately and oven dried at 70 °C for 48 h to obtain the dry biomass of each component. The two measurements were summed to derive the total seedling biomass.

### Biochemical analyses

Lipid peroxidation in the treated and untreated rice seeds was determined from the malondialdehyde (MDA) content using the thiobarbituric acid method^33^. The α-amylase activity in ground rice seeds (1 g) was measured according to the technique of Suksoon and Jaehyeun^34^. The rate of maltose liberation from starch was calculated from the reduction in 3,5-dinitrosalicylic acid. Total soluble sugars and soluble proteins were quantified according to Dubois *et al*.^35^ and Bradford^36^, respectively. The activities of superoxide dismutase (SOD) at 560 nm, CAT at 240 nm, POD at 470 nm, and GPx at 340 nm were determined according to Giannopolitis and Ries^37^, Dhindsa *et al*.^38^, Egley *et al*.^39^ and Drotar *et al*.^40^, respectively. One unit of enzyme activity was defined as that required to reduce 50% of nitro blue tetrazolium chloride per minute (SOD), oxidize 1 μmol H_2_O_2_ per minute (CAT), oxidize 1 μmol guaiacol per minute (POD), or catalyse the H_2_O_2_-induced oxidation of 1 μmol reduced glutathione at a pH of 7.0 and temperature of 25 °C (GPx). At 18 DAS, three randomly selected rice seedlings were harvested from each experimental unit. The total foliar soluble phenol content was determined from absorbance readings at 663 nm and 645 nm^41^. Photosynthetic pigments (chlorophyll a and b) were extracted in 80% v/v ice-cold acetone^42^ and read at 663 and 645 nm wavelengths.

### Statistical analyses

Data were analysed using the statistical software Statistix 8.1 (Analytical Software, Tallahassee, FL, USA), while differences among means were separated by using a least significant difference (LSD) test at the 5% probability level. Origin 8.1 (OriginLab Co., Northampton, MA, USA) was used for graphical representation.

## Data Availability

The data used to support the findings of this study are available from the corresponding author upon request.
